# Cannabinoid crystal polymorphism

**DOI:** 10.1186/s42238-022-00131-2

**Published:** 2022-04-17

**Authors:** Crist N. Filer

**Affiliations:** PerkinElmer Health Sciences Inc, 940 Winter Street, Waltham, MA 02451 USA

**Keywords:** *Cannabis*, Cannabinoid, Polymorph

## Abstract

Because cannabinoids are usually amorphous solids, the thought that some of them may also exist in distinctly different crystal polymorphic forms might at first seem unusual. However, this commentary provides compelling evidence and precedent for the likely existence of cannabinoid crystal polymorphism.

## Background

The structurally diverse substances comprising the ever-growing collection of cannabinoids (alternatively “phytocannabinoids”) found in *Cannabis* have been characterized by numerous spectroscopic tools over many decades (Radwan et al. 2021). One conspicuous omission in these methods has often been the examination of cannabinoid crystallinity and the reason for this is simple. After more than a century of cannabinoid investigation, most of them have been routinely described as low melting (usually less than 75 ^°^C) seemingly “amorphous” solids. Consequently, the proposal that any cannabinoid might exist in multiple crystal forms known as “polymorphs” would seem very unlikely. However, the purpose of this commentary is to challenge that perception and explore the possibility of cannabinoid crystal polymorphism.

## Crystal polymorphism

As mentioned, crystal polymorphism is defined as the existence of one or more distinctly different crystal forms of the same substance and it can manifest itself with both complex and uncomplicated molecules. Whatever the structural details, crystal polymorph creation is controlled by the intricate choreography of both nucleation kinetics and thermodynamic stability. Historically, it is commonly accepted that crystal polymorphism was first discovered for the very simple compound benzamide by the collaboration of noteworthy nineteenth century chemists Friedrich Wohler and Justus von Liebig in 1832. One can only imagine their amazement as they surprisingly observed the fine needles of one benzamide crystal polymorph convert to the rhombic crystals of another polymorph. Almost two centuries later, benzamide crystal polymorphism continues to fascinate current researchers (Kras et al. 2021). A myriad of crystal forms have now been characterized for many substances and thousands of them have been archived as X-ray crystal determinations in the voluminous Cambridge Structural Database (van de Streek 2006).

## Cannabinoid crystal polymorphism

**Fig. 1:**
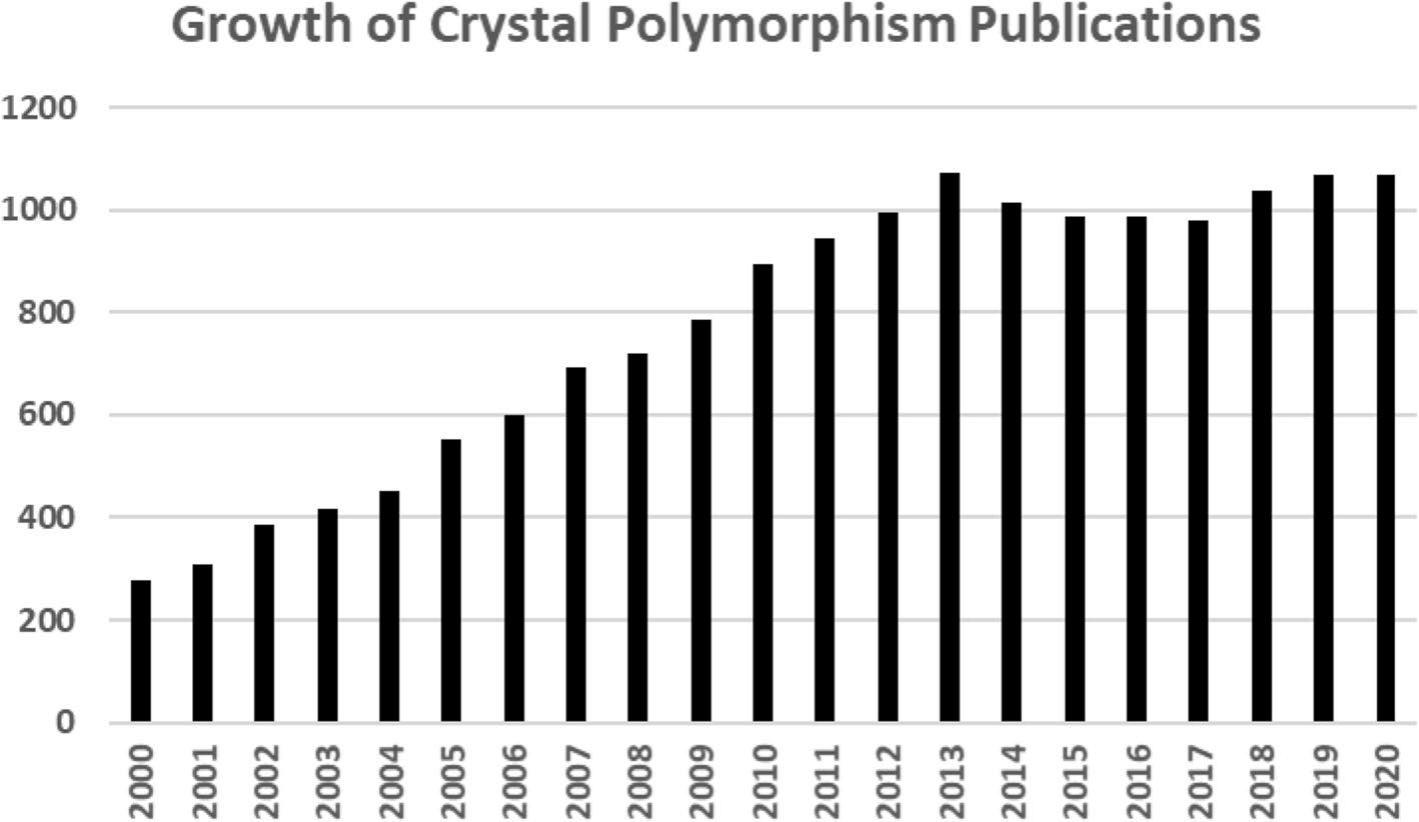
Growth of crystal polymorphism publications

**Fig. 2:**
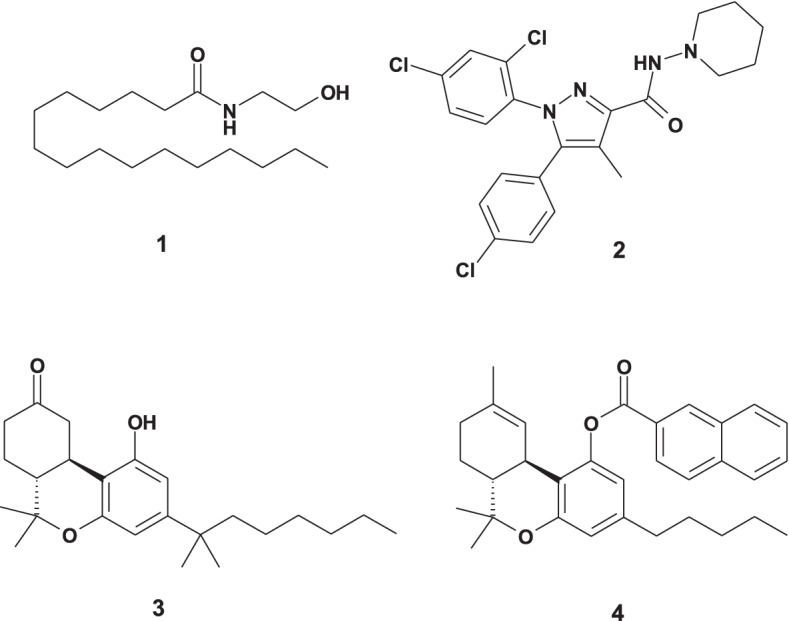
Cannabinoid associated substances exhibiting crystal polymorphism

Before addressing cannabinoid crystal polymorphism, it is useful to first consider which cannabinoids in *Cannabis* have even been analyzed as single crystalline entities? To answer this question, a literature search was performed using the Cambridge Structural Database as well as the SciFinder® chemistry database. Of the many cannabinoids in *Cannabis*, only a few have been characterized by X-ray crystal analysis and they include: delta-9-tetrahydrocannabinolic acid A (THCA-A) (Skell et al. 2021), delta-9-tetrahydrocannabinolic acid B (THCA-B) (Rosenqvist and Ottersen 1975), cannabidiol (CBD) (Mayr et al. 2017; Jones et al. 1977; Ottersen et al. 1977a), cannabinol (CBN) (Ottersen et al. 1977b), and cannabigerol (CBG) (Fettinger et al. 2020). Regarding polymorphism in general, it can be seen in Fig. 1 that growth of the crystal polymorphism literature (both journal articles and patents in SciFinder®) has dramatically increased, nearly quadrupling in the past 20 years. An exhaustive literature review was also performed to learn if any cannabinoid crystal polymorphs have been described? This search was conducted not only on the large number of publications captured under “cannabinoids” in general but also the very specific cannabinoids: THCA-A, delta-9-tetrahydrocannabinol (THC), cannabidiolic acid (CBDA), CBD, cannabigerolic acid (CBGA), CBG, cannabinolic acid (CBNA), CBN, cannabichromenic acid (CBCA), and cannabichromene (CBC). Unfortunately, this effort was greatly complicated by the many papers related to cannabinoid gene polymorphism. Excluding the special case of cocrystal polymorphs (Aitipamula et al. 2014) and any errors of omission due to the complexity of the literature search, it would appear that no fully characterized *Cannabis* cannabinoid crystal polymorph has yet been reported. However, there have been several intriguing clues in the literature that cannabinoid crystal polymorphism may well be possible (Fig. 2).

Interestingly, the cannabinoid receptors have been an important docking location for several structurally diverse compounds which have exhibited crystal polymorphism. The lipid *N*-palmitoylethanolamine [1] is an agonist at the type 2 cannabinoid receptor (CB-2). Two crystal polymorphs (designated α and β) of it were discovered and characterized by X-ray crystallography (Kamlekar and Swamy 2006). Also, the synthetic heterocycle rimonabant [2] is an inverse agonist at the type 1 cannabinoid receptor (CB-1) and has also demonstrated crystal polymorphism (Fours et al. 2015). However, even more compelling and direct evidence suggests that the basic cannabinoid structural framework itself has the capacity for crystal polymorphism as well. It was discovered 40 years ago that the synthetic 9-ketocannabinoid derivative nabilone [3] displayed crystal polymorphism. One of the earliest studies utilizing solid state carbon-13 NMR spectroscopy characterized several crystal polymorphic forms of nabilone (Byrn et al. 1985). Furthermore, the THC naphthoyl ester derivative [4] was just reported to exist in as many as eight different crystal polymorphic forms, designated A-H (Hallow et al. 2021). These separate crystal structures were analyzed by combinations of differential scanning calorimetry (DSC), thermal gravimetric analysis (TGA) as well as powder X-ray diffraction (PXRD). One crystal polymorph of 4 reportedly had a penchant for easily forming solvates and several of its crystal polymorphs were found to be interconvertible.

## Conclusions

Importantly, the discovery of cannabinoid crystal polymorphs would clearly be of more than mere academic interest. The very real possibility exists that such cannabinoid crystal polymorphs might possess greater stability or possibly improved bioavailability properties. It has been stated (Haleblian and McCrone 1969) that “probably every organic medicinal can exist in different polymorphs.” Also, in his recent X-ray crystal determination of THCA-A, internationally recognized Brandeis crystallographer Bruce Foxman speculated that this X-ray study with its important information about intermolecular cannabinoid hydrogen bonding would facilitate “the search for polymorphs” (Skell et al. 2021).

Based on the foregoing evidence, it is reasonable to conclude that at least some *Cannabis* cannabinoids will also exhibit crystal polymorphism.

## Data Availability

Not applicable,

## Abbreviations

CBCA: Cannabichromenic acid
CBC: Cannabichromene
CBDA: Cannabidiolic acid
CBD: Cannabidiol
CBGA: Cannabigerolic acid
CBG: Cannabigerol
CBNA: Cannabinolic acid
CBN: Cannabinol
CB-1: Cannabinoid receptor type 1
CB-2: Cannabinoid receptor type 2
DSC: Differential scanning calorimetry
NMR: Nuclear magnetic resonance
PXRD: Powder X-ray diffraction
TGA: Thermal gravimetric analysis
THCA-A: Delta-9-tetrahydrocannabinolic acid A
THCA-B: Delta-9-tetrahydrocannabinolic acid B
THC: Delta-9-tetrahydrocannabinol
